# Prevalence of mycoplasma-like lung lesions in pigs from commercial farms from Spain and Portugal

**DOI:** 10.1186/s40813-021-00204-3

**Published:** 2021-03-08

**Authors:** FJ Pallarés, JA Añón, IM Rodríguez-Gómez, J Gómez-Laguna, R Fabré, JM Sánchez-Carvajal, I Ruedas-Torres, L Carrasco

**Affiliations:** 1 Department of Anatomy, Comparative Pathology and Toxicology, Faculty of Veterinary Medicine, University of Córdoba, 14014 Córdoba, Spain; 2 Ecuphar Veterinaria SLU, 08016 Barcelona, Spain; 3 Present address: Olmix Ibérica SLU, 31192 Mutilva, Navarra, Spain; 4 Present address: Forestal Catalana SA, 08012 Barcelona, Spain

**Keywords:** *Mycoplasma hyopneumoniae*, Lung lesions, Prevalence, Histopathology, Scoring system

## Abstract

**Background:**

*Mycoplasma hyopneumoniae* causes a chronic respiratory disease that produces important economic losses due to poor productive performance, increased mortality and costs for several control strategies. The prevalence of mycoplasma-like lesions (MLL) at abattoir has been widely studied in different countries, making use of different scoring systems. However, most of them are difficult to apply in abattoirs with high number of pigs sacrificed per hour. For that reason, it is necessary to adapt the scoring system to the reality of the modern abattoir, even if there is a loss of accuracy. Our purpose was to investigate the prevalence and severity of MLL at abattoirs in Spain and Portugal using a 0 to 5 scoring system adapted to abattoirs with high number of sacrificed pigs per hour and to highlight the histopathological diagnosis as confirmatory method to identify patterns of pneumonia correlated to gross lesions.

**Results:**

Cranioventral pulmonary consolidation, a typical MLL, was the most frequent lung lesion (30.97 %) detected at the abattoir, followed by dorsocaudal infarcts with pleurisy (12.51 %) and pleurisy alone (6.26 %). The average score for all examined lungs at abattoir was 1.99 out of 5 points. The histopathological study revealed that the 78.17 % of the randomly selected lungs with MLL presented microscopic lesions compatible with *M. hyopneumoniae* infection. Most bronchointerstitial and interstitial pneumonia lesions had a chronic course while most suppurative and fibrinous bronchopneumonia lesions had an acute course and a higher degree of severity. The combination of microscopic lesions more frequently observed was bronchointerstitial pneumonia + interstitial pneumonia + suppurative bronchopneumonia.

**Conclusions:**

The prevalence of MLL at abattoir was 30.97 %, however, after microscopic examination the real prevalence of lungs with lesions compatible with *M. hyopneumoniae* infection was reduced up to 24.21 %. The six more prevalent combinations of lesions in the microscopic study involved the 66.13 % of examined lungs, and in all of them, microscopic lesions characteristic of *M. hyopneumoniae* infection were found, what supports the importance of *M. hyopneumoniae* as a primary pathogen in cases of PRDC.

## Background

*Mycoplasma hyopneumoniae* is the primary etiological agent of enzootic pneumonia (EP), a chronic respiratory disease considered as one of the most widespread and economically damaging diseases for the swine industry [1–3]. *M. hyopneumoniae* plays a pivotal role as primary agent of porcine respiratory disease complex (PRDC), a multifactorial disease resulting from the interaction of different infectious agents (viruses, mycoplasmas and bacteria), management and environmental conditions and host factors [4, 5]. Typical EP gross lung lesions are characterized by well defined, greyish to reddish depressed cranioventral areas of consolidation. Microscopically, these areas correspond with a pattern of bronchointerstitial pneumonia with lymphoid cells infiltrating the lamina propria of bronchioles to a differing extent and, finally, evolving a hyperplasia of bronchus-associated lymphoid tissue (BALT) at peribronchial, peribronchiolar and perivascular levels [6–8].

*Post-mortem* inspection at abattoir is a truly common and useful practice in many countries to gather information about herd health status and to monitor the efficacy of treatments and management conditions. Prevalence and severity of lung lesions, identification of the possible etiology and herd risk factors associated with these lesions, as well as the impact of the lesions on performance indicators and carcass and meat quality have been the subject of many studies [9–16]. Thus, a high variation among countries in the prevalence of mycoplasma-like lesions (MLL) at abattoir, ranging from 23.85 % in Belgium to 72.60 % in Germany, has been reported [9, 11, 12, 15, 17–20].

Several and different methods can be performed for evaluating the severity of lung lesions at abattoir [21–25]. Most of them are based on the quantification of the affected lung surface [22–25], nevertheless, nowadays, due to the speed of the slaughterline at industrial abattoirs, with more than 500 pigs per hour being slaughtered, some of these methods are impractical and difficult to follow. For that reason, the scoring systems need to be modified and adapted to the new reality, trying to be easy to perform and repeatable, even if there is a tiny loss of accuracy.

The association of lung lesions at abattoir, such as pleurisy and lung scars, with poor performances as decreased average daily gain (ADG) and economic return [16, 26] highlights the importance of monitoring lesions at abattoir. However, the prevalence of microscopic lung lesions has been reported to be much higher than the prevalence of macroscopic ones when investigating non-infectious factors associated with gross and microscopic lung lesions in slaughtered pigs [9]. In this sense, whereas gross lesions, such as MLL or pleurisy, can take between 8 and 12 weeks to heal and disappear [6, 7, 27], microscopic lesions may persist for longer [9]. Thus, histopathology can bring to light the presence of lesions that grossly can go unnoticed.

The aim of this study was to investigate the prevalence and severity of MLL at abattoirs in Spain and Portugal using a 0 to 5 scoring system adapted to a high number of slaughtered pigs per hour, to know the real prevalence of lesions associated with *M. hyopneumoniae* infection as well as to highlight the use of histopathology to confirm the lesions and identify other patterns involved in the examined lungs.

## Results

### Lung examination at abattoir and scoring

**Fig. 1:**
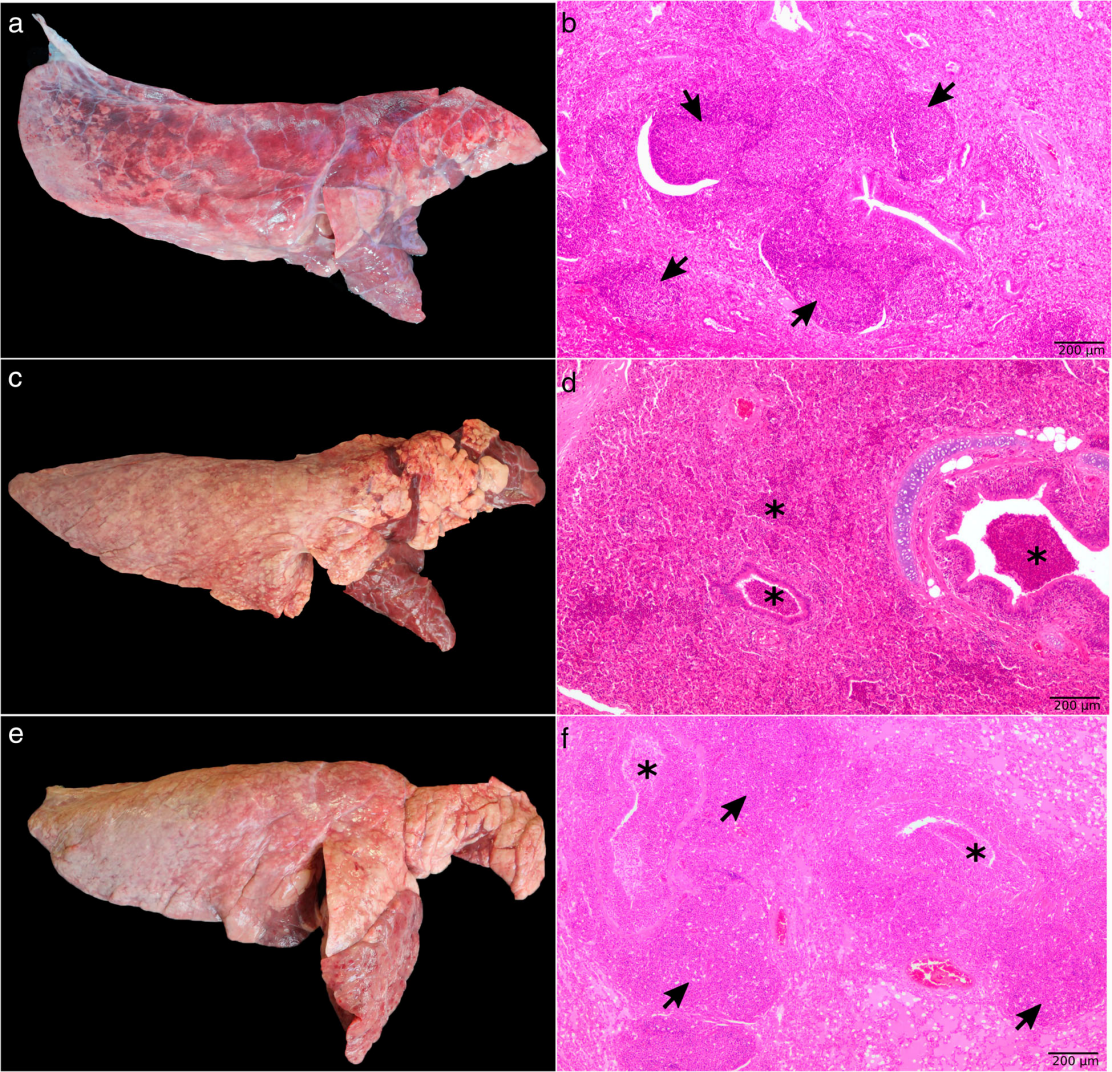
Lungs showing Mycoplasma-like lesions at abattoir (**a**, **c** and **e**) and their corresponding microscopic lesions (**b**, **d** and **f**, respectively). **b**: Peribronchiolar lymphoid clumps of cells of varying degrees of development infiltrating the lamina propria of the bronchioles to a differing extent (BALT hyperplasia) (arrows). Bronchointerstitial pneumonia. **d**: Exudate inside bronchi, bronchioles and alveoli, predominantly composed of degenerated neutrophils (asterisks). Suppurative bronchopneumonia. **f**: Mixed pattern of lesions showing both types of pneumonia described in pictures **b** and **d** (arrows and asterisks). Bronchointerstitial pneumonia + suppurative bronchopneumonia

The type and percentage of lung lesions observed at abattoir are showed in Table 1. Approximately half of all examined lungs at abattoir did not exhibit any gross lung lesion (100,371 lungs; 50.26 %). Cranioventral pulmonary consolidation (compatible with MLL) was the most frequent lung lesion (61,832 lungs; 30.97 %) (Fig. 1a, c and e), followed by dorsocaudal infarcts with pleurisy (compatible with *Actinobacillus pleuropneumoniae* infection) (24,970 lungs; 12.51 %) and pleurisy alone, that was recorded in 12,505 lungs (6.26 %). Similar percentages of MLL were found in Spain (31.14 %) and in Portugal (29.95 %).

**Table 1:** Type, number and percentage of gross lesions observed at abattoir. In case of cranioventral pulmonary consolidation, the number and percentage of lungs belonging to each score (1–5) has been itemized

Gross lung lesion	Number of lungs (percentage)
Cranioventral pulmonary consolidation	61,832 (30.97 %)
* Score 1*	*26,046 (13.04 %)*
* Score 2*	*18,008 (9.02 %)*
* Score 3*	*11,408 (5.71 %)*
* Score 4*	*4,925 (2.47 %)*
* Score 5*	*1,445 (0.72 %)*
Dorsocaudal infarcts with pleurisy	24,970 (12.51 %)
Pleurisy alone	12,505 (6.26 %)
No lesion	100,371 (50.26 %)

Table 1 includes the number of lungs according to the given score for MLL. Scores 1 and 2 were the most frequent with 13.04 % (26,046 lungs) and 9.02 % (18,008 lungs), respectively. The average score of all lungs exhibiting cranioventral consolidation was 1.99, no finding differences across countries, 2.00 for Spain and 1.93 in the case of Portugal.

### Histopathology of the lungs

As depicted in Table 2, bronchointerstitial pneumonia, compatible with *M. hyopneumoniae* infection, was the most frequent lesion pattern (78.17 %) (Fig. 1b), followed by suppurative bronchopneumonia (73.47 %) (Fig. 1d), interstitial pneumonia (68.65 %) and fibrinous bronchopneumonia (14.21 %). The highest degree of severity was observed in cases of fibrinous bronchopneumonia (2.89), followed by suppurative bronchopneumonia (2.52), bronchointerstitial (2.17) and interstitial pneumonia (1.74), the latter with the lowest score. With respect to the course, most of the cases of bronchointerstitial and interstitial pneumonia had a chronic course, whereas most of the cases of suppurative and fibrinous bronchopneumonia had an acute course. Moreover, roughly 41 % of the studied lungs showed pleurisy, chronic in most of the cases (89.17 %).

**Table 2:** Number, severity and course for each histopathological pattern of pneumonia

Type of microscopic lesion	Number (%)	Severity (1–3)	Course (acute/chronic)
Bronchointerstitial pneumonia	616 (78.17 %)	2.17	(3.09 % / 96.91 %)
Suppurative bronchopneumonia	579 (73.47 %)	2.52	(72.36 % / 27.64 %)
Fibrinous bronchopneumonia	112 (14.21 %)	2.89	(66.07 % / 33.93 %)
Interstitial pneumonia	541 (68.65 %)	1.74	(3.51 % / 96.49 %)
Pleurisy	323 (40.99 %)	-	(10.83 % / 89.17 %)

The more prevalent combinations of microscopic patterns of lesions are showed in Table 3. Of note, more than one lesion pattern coexisted in most sampled lungs. Thus, from the 788 microscopically examined lungs, 720 (91.37 %) presented more than one microscopic pattern, 59 lungs (7.49 %) only showed one microscopic pattern and, only 9 lungs (1.14 %) did not exhibit any microscopic lesion. The most frequent combination of microscopic patterns was bronchointerstitial pneumonia + interstitial pneumonia + suppurative bronchopneumonia in 171 lungs (21.70 %) and the same combination accompanied by pleurisy in other 108 lungs (13.71 %), followed by the combination of bronchointerstitial pneumonia + suppurative bronchopneumonia (Fig. 1f) found in 77 lungs (9.77 %) and in 62 additional lungs together with pleurisy (7.87 %). The six more prevalent combinations of microscopic patterns of pneumonia represented two thirds (66.13 %) of the lungs microscopically examined, with lesions characteristic of *M. hyopneumoniae* infection being found in all of them (Table 3). Combinations with an equal number of lesion patterns showed a higher degree of severity when bronchopneumonia was present, irrespective of the type of bronchopneumonia.

**Table 3:** More prevalent combinations of microscopic patterns of pneumonia including the number and percentage of lungs and lesion severity

Patterns of microscopic lesion	Number (%)	Severity
Bronchointerstitial pneumonia + interstitial pneumonia + suppurative bronchopneumonia	171 (21.70 %)	6.44
Bronchointerstitial pneumonia + interstitial pneumonia + suppurative bronchopneumonia + pleurisy	108 (13.71 %)	7.45
Bronchointerstitial pneumonia + suppurative bronchopneumonia	77 (9.77 %)	4.64
Bronchointerstitial pneumonia + suppurative bronchopneumonia + pleurisy	62 (7.87 %)	5.55
Bronchointerstitial pneumonia + interstitial pneumonia	53 (6.73 %)	4.11
Bronchointerstitial pneumonia + interstitial pneumonia + pleurisy	50 (6.35 %)	5.12
Suppurative bronchopneumonia + interstitial pneumonia	40 (5.08 %)	4.18
Interstitial pneumonia	23 (2.92 %)	1.83

## Discussion

Despite the reported efficacy of *M. hyopneumoniae* vaccines in reducing lung lesions [28] and bacterial load from the respiratory tract [29], they are not capable of fully eliminate the bacteria from the animal, giving them the opportunity to cause typical MLL in a variable percentage of pigs at abattoir in different countries [12, 17, 19, 20]. To investigate the prevalence and severity of MLL at abattoirs in Spain and Portugal using a 0 to 5 scoring system adapted to fast slaughterlines, a study with an elevated number of pigs (199,678), involving a total of 994 batches from 221 different farms, was performed. Additionally, a sample of 788 lungs was taken to corroborate microscopically the presence of lesions characteristics of the infection induced by *M. hyopneumoniae.*

In our study, no gross lesions were found in 50.26 % of all examined lungs at abattoir and roughly 31 % showed cranioventral consolidations compatible with mycoplasma lesions, showing similar frequencies in Spain (31.14 %) and Portugal (29.95 %). These prevalence rates of MLL were higher than those reported in Belgium (23.85 % and 24.00 %) [9, 17] but lower than those previously found in Spain (44.61 to 55.69 %) [11, 19], Italy (46.38 %) [12], France (69.30 %) [18] or Germany (72.60 %) [20]. The main difference among studies was the number of examined lungs, with nearly 200,000 lungs evaluated in this study in comparison with numbers ranging from 600 [19] to 10,404 in other studies [11]. However, other factors such as the epidemiological scenario in each country also play a role in these differences. Consequently, considering the high number of batches from different farms and the different pig production areas herein reported, we consider that our data provide an accurate percentage of prevalence of MLL in Spain and Portugal. Moreover, if we consider the histopathological results and the percentage of selected lungs with MLL compatible with *M. hyopneumoniae* infection (78.17 %), the real prevalence of gross lesions caused by this bacterium would be reduced from 30.97 % to 24.21 %. Other lesions, such as suppurative bronchopneumonia, as represented in Fig. 1, exhibited a similar gross appearance [30, 31] and, hence, this type of pneumonia could be mixed up with lesions caused by *M. hyopneumoniae*. Thereby, those studies, in which a microscopic confirmation has not been performed, could bias the results, overestimating the prevalence. In the study carried out by Luhers et al. [20], 78.30 % of 400 lungs with MLL collected at the abattoir were positive to *M. hyopneumoniae* by PCR, a percentage very similar to the one found with compatible microscopic lesions in our study (78.17 %). These results highlight the interest of performing additional studies on lungs with MLL to confirm the diagnosis of EP or infection by *M. hyopneumoniae*.

Pleurisy, associated with dorsocaudal infarcts or alone, was the second most prevalent gross lesion of our study, affecting 18.77 % of the examined lungs, a percentage very similar to that found in France (15.00 %) [18] or Belgium (16.00 %) [9], but lower than those previously reported in Italy (25.10 %) [12] or Spain (26.80 %) [11]. In our case, most of the pleurisy (66.65 %) was associated with dorsocaudal infarcts, a hallmark of *A. pleuropneumoniae* infection, lesion which has been associated with decreased ADG during the grower-finisher period, leading to lower economic return [16]. Therefore, the measures to control this pathogen (management, therapeutic or vaccination) should be revised and/or implemented in the farms included in the study.

According to our findings, most of the lesions of bronchointerstitial and interstitial pneumonia, characteristics of mycoplasma and viral infections, respectively, had a chronic course, by contrast, the majority of the lesions of suppurative and fibrinous bronchopneumonia, characteristics of bacterial infection, had an acute course and a higher degree of severity. These results suggest that viruses and mycoplasmas could have acted in an earlier stage of the life of piglets, probably during nursery, and later on, during the fattening period, bacteria would have taken action. Ruggeri et al. [32] reported pleurisy, followed by pleuropneumonia, catarrhal bronchopneumonia and bronchointerstitial pneumonia, as the most prevalent microscopic lesions in fattening pigs, but animals included in that study died because of respiratory diseases, that is, samples were not collected from healthy animals at abattoir as in the present study. However, to a certain extent, it agrees with our observations, since most of the deaths were consequence of an acute process of fibrinous and suppurative bronchopneumonia. A recent study carried out in Brazil by Galdeano et al. [15] also found the characteristic lesions of *M. hyopneumoniae* infection as the most prevalent one, being detected in 63.75 % of the lungs examined microscopically, but different to our study, the second most frequent lesion was chronic bronchopneumonia (57.14 %) whereas other lung lesions common in our study such as suppurative bronchopneumonia, interstitial pneumonia or fibrinous bronchopneumonia, were observed in a lesser extent (15.63 %, 3.61 % and 0.15 %, respectively). Nevertheless, it must be taken into account that porcine reproductive and respiratory syndrome virus (PRRSV) has never been detected in Brazil [33], thus, one of the main primary agents involved in the PRDC [5], alone or in combination with other pathogens, is not taken place in those farms. Therefore, the clinical and lesional picture at abattoir would be totally different in Brazil, when compared with those countries where PRRSV is endemic, such as Spain or Portugal.

The histopathological study provided us a more accurate idea of the real prevalence of lesions caused by *M. hyopneumoniae* but also information about the concomitant action of other pathogens involved in the PRDC that sometimes go clinically unnoticed. In addition, some lesions are unspecific and could be caused by different pathogens, but in association with other techniques, such as serology, bacteriology or PCR, histopathology could provide a more accurate information about which agent is causing the lesion, since a positive result in those diagnostic techniques against some pathogens [i.e., *M. hyopneumoniae* or porcine circovirus type 2 (PCV2)] does not necessarily always mean that they are causing any lesion.

Lesions of bronchointerstitial pneumonia compatible with *M. hyopneumoniae* infection were found in the six more frequent combinations of lesion patterns in our study, representing 66.13 % of the lungs examined, what support the relevant role of this agent in the PRDC as a primary agent as well as enhancing the action of other pathogens involved in as PRRSV [34], PCV2 [35] or swine influenza virus [36].

The scoring system from 0 to 5 points used in this study for MLL, adapted from a previous one [37], has been showed as a simple and repeatable method that can be easily applied in cases of abattoirs with fast slaughterline, sometimes more than 500 pigs per hour. By contrast, it is not as precise as other methods which express the proportion of affected lung area in percentages [22–25].

## Conclusions

The 0 to 5 points scoring system proposed in the present study showed to be a simple, useful and repeatable method that can be easily conducted in cases of abattoirs with a fast slaughterline. An average score of MLL of 1.99 was obtained by using this scoring system and a real prevalence of 24.21 % was determined after confirmation of cranioventral consolidation compatible with mycoplasma lesions by histopathology. Our results highlight the significance of *M. hyopneumoniae* in PRDC, acting as a primary agent in combination with other pathogens which lead to different patterns of lung lesions, and emphasize the necessity of implementing holistic control measures against this agent.

## Methods

### Study population

A total of 199,678 pigs from 221 different herds from Spain (170,174 pigs; 85.22 %) and Portugal (29,504 pigs; 14.78 %) were examined at abattoir from 2013 to 2017. Herds with different production systems (one-site, two-sites or three-sites) and size (between 200 and 8,000 sows) were included in the study. The number of fattening units per herd varied between one and eight with a total of 994 units being included. All herds herein included belonged to the *Mycoguard Program*, a project conducted by Ecuphar Veterinaria SLU with the purpose of evaluating the incidence and type of lung lesions at abattoir, with special focus on MLL. A batch from each fattening unit of the herd (average size of 200 pigs) was examined at the abattoir. The first and the last batches to be sent to the abattoir in each fattening unit were excluded to avoid the best and the worst performing pigs of the unit. The average weight and age of the pigs at sacrifice were 100 kg (95–105 kg) and 6 months, respectively. Most of the origin farms (142/221; 64.25 %) were located in southern Spain (regions of Andalusia, Extremadura and Murcia), followed by 25.79 % (57/221) in the north of Spain (regions of Cataluña, Aragón and Castilla y León) and 9.96 % (22/221) in Portugal. All considered farms have historically vaccinated against *M. hyopneumoniae* with commercial vaccines.

### Lung examination at abattoir and scoring

Lung examination was performed in 46 different abattoirs located in Spain (36) and Portugal (10) by seven veterinarians, which had been trained by three different specialists in swine pathology to follow the same scoring criteria in order to avoid individual discrepancies, although no formal assessment was made of the agreement between the veterinarians. The slaughterline speed ranged from 350 to 600 pigs per hour, but in most of the abattoirs the speed was comprised between 500 and 600 pigs per hour. All pigs were stunned with CO_2_ before bleeding. Lungs were visually appraised and palpated to detect lesions compatible with pneumonia. The system used for scoring cranioventral consolidations in the lung was based on the previously score proposed by Bollo et al. [37] with some modifications. Briefly, score 0: no lesion observed; score 1: consolidation affecting unilaterally the apex of one or two different lung lobes; score 2: consolidation affecting bilaterally the apex of one or two different lung lobes; score 3: consolidation affecting bilaterally the apex and medial part of one or two different lung lobes; score 4: consolidation affecting bilaterally the apex and medial part of one or two different lung lobes and partial involvement of the cranial area of one caudal lung lobe; score 5: consolidation affecting all lobes, including the cranial area of both caudal lobes. The average score was calculated for each batch (data not showed) and each country. The presence of other lesions, such as infarcts, abscesses and pleurisy, was also recorded.

### Histopathology of the lung

Three to four lungs per batch with MLL were randomly selected at the abattoir for histopathological examination. Therefore, a total of 788 lungs, 737 from Spain and 51 from Portugal, were pictured and four samples per lung were collected: one belonging to the cranial lung lobe, other from the middle lung lobe and two from the ventral and dorsal areas of the caudal lung lobe of the right lung. In case of the left lung, the two portions of the cranial lung lobe together with the two samples coming from the caudal lung lobe were collected. The specimens were collected at the boundary junction of affected and unaffected tissue. Samples were fixed in 10 % neutral buffered formalin for 24 hours, embedded in paraffin wax, sectioned at 4 microns and stained with hematoxylin and eosin.

Microscopic lung lesions of pneumonia were classified as bronchopneumonia (suppurative or fibrinous), bronchointerstitial and interstitial pneumonia according to the morphological pattern. Briefly, bronchopneumonia was characterized by the presence of inflammatory exudate into bronchi, bronchioles and alveoli that in the case of suppurative bronchopneumonia was predominantly composed of degenerated neutrophils while in fibrinous bronchopneumonia the predominant component of the exudate was fibrin, associated with the presence of necrosis and hemorrhages. Interstitial pneumonia was characterized by the thickening of alveolar walls by the presence of mononuclear cells and hyperplasia and hypertrophy of type II pneumocytes. In the case of bronchointerstitial pneumonia, mononuclear cells encircling airways and infiltrating alveolar septa and BALT hyperplasia were present [30, 31]. Pleurisy, characterized by the presence of fibrin or connective tissue in the pleura, was also recorded. Each microscopic lesion was scored according to its severity in mild (score 1), moderate (score 2) or severe (score 3), except for pleurisy that was scored as 0 (absence) or 1 (presence). The criteria for the scoring of microscopic lung lesions are summarized in Table 4. The microscopic score was calculated for each pattern of pneumonia and the final score was calculated by adding the individual scores for each type of pneumonia. As four samples were microscopically examined per lung, the score selected for each lesion pattern was the most severe one observed in any of the examined samples.

**Table 4:** Scoring system used to determine the severity of microscopic lung lesions

Type of pneumonia	Mild (Score 1)	Moderate (Score 2)	Severe (Score 3)
**Bronchointerstitial pneumonia**	BALT in > 5 bronchi or bronchioles (1–2 BALT per structure)	**1.-** BALT in > 5 bronchi or bronchioles (50 % of the structure is surrounded by BALT) or **2.-** BALT in > 5 bronchi or bronchioles (1–2 BALT per structure) and one bronchus or bronchiole completely surrounded by hyperplasia of BALT	**1.-** At least two structures are almost completely surrounded (80 %) by hyperplasia of BALT or **2.-** All structures of the section are affected
**Interstitial pneumonia**	1–2 lobule/s affected^a^	50 % affected^a^	All lobuli are affected^a^
**Suppurative pneumonia**	1–2 lobule/s affected^a^	50 % affected^a^	All lobuli are affected^a^
**Fibrinous pleuropneumonia**	1–2 lobule/s affected^a^	50 % affected^a^	All lobuli are affected^a^
**Pleurisy**	Presence	---	---

The term “structure” describes a bronchus or bronchiole, equally

*BALT* bronchus-associated lymphoid tissue

^a^Every evaluated section included at least 6 lobuli

Lesions were also differentiated according to their course as acute or chronic lesions. Acute lesions were defined as those with neutrophils as the dominant inflammatory cell type, extensive edema and fibrin exudation. Chronic lesions were characterized by mononuclear cell infiltrate, primarily consisting of lymphocytes and plasma cells, proliferation of connective tissue, epithelial or BALT hyperplasia, and hypertrophy of the smooth muscle layer around bronchioles and alveolar ducts.

All the slides were blindly evaluated by two pathologists to determine the pattern and score of pneumonia.

## Data Availability

The datasets used and analyzed during the current study are available from the corresponding author on reasonable request.

## Abbreviations

ADG: Average daily gain
BALT: Bronchus-associated lymphoid tissue
EP: Enzootic pneumonia
MLL: Mycoplasma-like lesions
PCV2: Porcine circovirus type 2
PRDC: Porcine respiratory disease complex
PRRSV: Porcine reproductive and respiratory syndrome virus
